# Analytical Performance Evaluation of New DESI Enhancements for Targeted Drug Quantification in Tissue Sections

**DOI:** 10.3390/ph15060694

**Published:** 2022-06-01

**Authors:** Margaux Fresnais, Siwen Liang, Marius Breitkopf, Joshua Raoul Lindner, Emmanuelle Claude, Steven Pringle, Pavel A. Levkin, Konstantin Demir, Julia Benzel, Julia Sundheimer, Britta Statz, Kristian W. Pajtler, Stefan M. Pfister, Walter E. Haefeli, Jürgen Burhenne, Rémi Longuespée

**Affiliations:** 1 Department of Clinical Pharmacology and Pharmacoepidemiology, Heidelberg University Hospital, Im Neuenheimer Feld 410, 69120 Heidelberg, Germany; margaux.fresnais@med.uni-heidelberg.de (M.F.); siwen.liang917@gmail.com (S.L.); breitkopf@stud.uni-heidelberg.de (M.B.); joshua.lindner@stud.uni-heidelberg.de (J.R.L.); walter-emil.haefeli@med.uni-heidelberg.de (W.E.H.); juergen.burhenne@med.uni-heidelberg.de (J.B.); 2 Waters, Stamford Avenue, Altrincham Road, Wilmslow SK9 4AX, UK; emmanuelle_claude@waters.com (E.C.); steven_pringle@waters.com (S.P.); 3 Aquarray, Hermann-von-Helmholtz-Platz 6, 76344 Eggenstein-Leopoldshafen, Germany; pavel.levkin@aquarray.com (P.A.L.); konstantin.demir@aquarray.com (K.D.); 4 Institute of Biological and Chemical Systems-Functional Molecular Systems (IBCS-FMS), Karlsruhe Institute of Technology (KIT), Hermann-von-Helmholtz-Platz 1, 76344 Eggenstein-Leopoldshafen, Germany; 5 Hopp Children’s Cancer Center Heidelberg (KiTZ), Im Neuenheimer Feld 430, 69120 Heidelberg, Germany; j.benzel@kitz-heidelberg.de (J.B.); j.sundheimer@kitz-heidelberg.de (J.S.); b.statz@dkfz-heidelberg.de (B.S.); k.pajtler@kitz-heidelberg.de (K.W.P.); s.pfister@kitz-heidelberg.de (S.M.P.); 6 Division of Pediatric Neurooncology, German Cancer Research Center (DKFZ), German Cancer Consortium (DKTK), Im Neuenheimer Feld 280, 69120 Heidelberg, Germany; 7 Department of Pediatric Hematology, Oncology and Immunology, Heidelberg University Hospital, 69120 Heidelberg, Germany

**Keywords:** desorption electrospray ionization, mass spectrometry, drug, quantification, profiling

## Abstract

Desorption/ionization (DI)-mass spectrometric (MS) methods offer considerable advantages of rapidity and low-sample input for the analysis of solid biological matrices such as tissue sections. The concept of desorption electrospray ionization (DESI) offers the possibility to ionize compounds from solid surfaces at atmospheric pressure, without the addition of organic compounds to initiate desorption. However, severe drawbacks from former DESI hardware stability made the development of assays for drug quantification difficult. In the present study, the potential of new prototype source setups (High Performance DESI Sprayer and Heated Transfer Line) for the development of drug quantification assays in tissue sections was evaluated. It was demonstrated that following dedicated optimization, new DESI XS enhancements present promising options regarding targeted quantitative analyses. As a model compound for these developments, ulixertinib, an inhibitor of extracellular signal-regulated kinase (ERK) 1 and 2 was used.

## 1. Introduction

Desorption/ionization (DI)-mass spectrometry (MS) can now be realistically considered for the development of reliable methods for drug quantification in biological matrices and their validation following regulatory guidelines [1]. Although in some contexts, direct on-surface analysis of solid or solidified samples suffers from the lack of chromatographic separation of target compounds from endogenous species in biological matrices, this can be partially compensated by using high-resolution MS measurements and/or further post-ionization gas-phase separation using ion mobility (IM)-MS [2,3,4]. Moreover, DI-MS methods offer a considerable advantage of rapidity and low-sample input for drug quantification. DI-MS methods have extensively been used for the quantification of drugs in fluids [5,6,7,8,9,10,11], recently supported by full method validation [11]. In addition to the advantage of rapidity, DI-MS of drugs in tissue sections preserves histological contexts and can be performed at different dimensional scales, from profiling (when regions of interest are measured) [1,2,12] to imaging (when a complete drug mapping is necessary) [13,14,15,16,17,18,19,20,21,22,23,24,25,26,27,28,29,30,31,32,33,34,35,36,37,38,39,40]. Different development and validation approaches were also proposed for the quantification of drugs in tissue sections using DI-MS profiling and imaging [1]. Validation was already approached by different groups, using matrix-assisted laser desorption ionization (MALDI) [2,38]. Atmospheric pressure and desorption matrix-free DI-MS sources such as desorption electrospray ionization (DESI) theoretically offer additional advantages of (i) more direct and therefore even more rapid analyses due to the absence of delays to vent the ion source before analysis, (ii) reduced chemical interference due to the absence of desorption matrices as in MALDI [41], and (iii) reduced ion suppression effects induced by desorption matrices [42]. Since the introduction of DESI as an ion source for DI-MS, improvements have been made in terms of emitter positioning [43], ion generation/ion injection synchronization [43], geometry of the solvent capillary [44], or coupling of primary/nanospray capillaries (nano-DESI) [45], to cite a few. Practically, few commercial options are available for universal use and former DESI-MS hardware [44] suffered from a poor robustness for the development of drug quantification assays, due to the fragility of the glass emitters [46]. In the present study, the potential of new DESI enhancements, i.e., a High-Performance (HP) Sprayer available for DESI XS and a Heated Transfer Line, were evaluated for the development of drug quantification assays in tissue sections. The extracellular signal-regulated kinase (ERK) 1 and 2 inhibitor ulixertinib (ULN) was used as a model compound for analytical evaluation in mouse brain tissue sections. Current in vitro and in vivo assays performed in our working group indicate that ULN holds good promise for the treatment of pediatric low-grade gliomas. The penetration of the drug in the mouse brain was demonstrated and its concentration correlated with on-target activity (loss of ERK phosphorylation). These findings made ULN a good model for these developments. The results indicate that the HP DESI Sprayer offers the necessary requirements for the development of assays for drug quantification in tissue sections according to regulatory guidelines.

## 2. Results

### 2.1. Cone Voltage and Ion Transfer Tube Temperature Optimization

**Figure 1:**
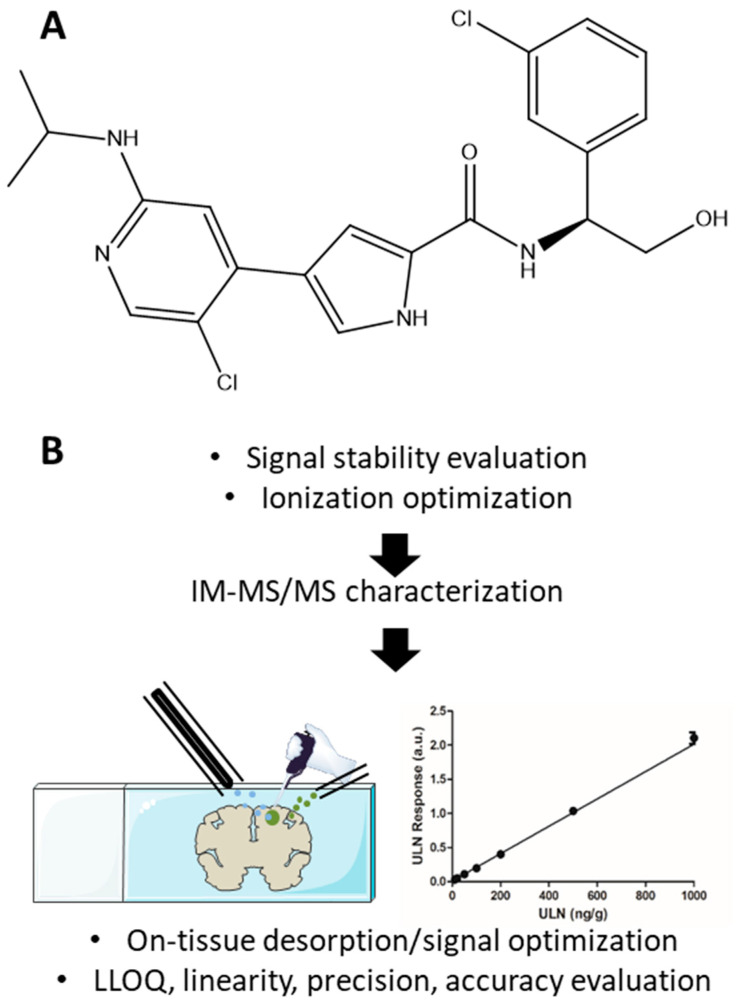
(**A**) Chemical structure of ulixertinib. (**B**) Steps for analytical performance evaluation.

Two prototypes were evaluated for the development of quantitative DESI-MS assays, using as a model the ERK inhibitor ULN (Figure 1A): a HP DESI sprayer and a Heated Transfer Line (see Section 4 and Section 4.6). The first phase of method development consisted of signal stability evaluation and ionization optimization (Figure 1B, see Section 4, Section 4.3, Section 4.5 and Section 4.6).

**Figure 2:**
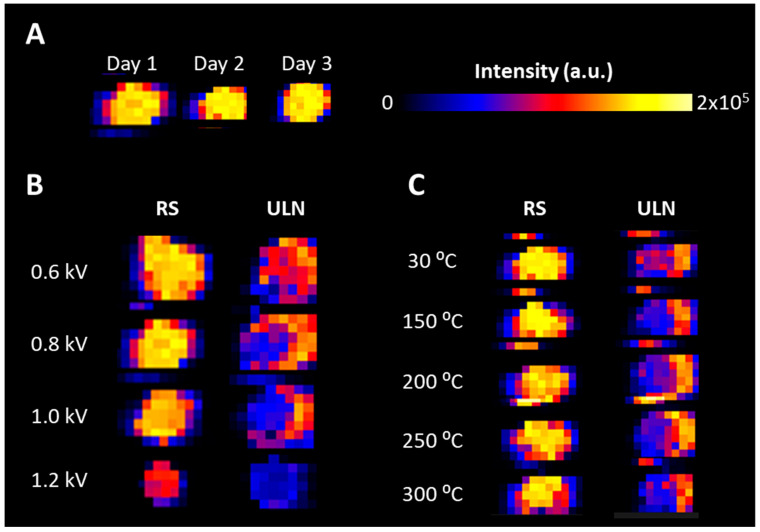
**Optimization of the initial settings for DESI-MS analyses.** Rhodamine C from a red staining marker (RS, *m*/*z* 443) and ulixertinib (ULN, *m*/*z* 433) were deposited on Aquarray superhydrophilic glass slides. (**A**) Signal stability was compared between images of RS acquired on three different days. (**B**) Different voltages were compared for RS and ULN analysis, both displaying the highest signal for a capillary voltage of 0.8 kV. (**C**) Different temperatures were tested for RS and ULN analysis, both displaying equal signals with any temperature.

As the capillary voltage is a critical parameter for signal improvement, different values were tested within the limits evaluated during the development of the hardware (i.e., <1.2 kV). Droplets of ULN solution deposited on Aquarray slides were analyzed. Because the solvent spray could rapidly solvate and “wash-off” the deposit, the substance may rapidly disappear at different kinetics between spots if a direct profiling [47] was used, thus preventing any possible relative signal comparisons. Therefore, the imaging-MS mode was used for these tests. As a control of sprayer positioning, desorption, and signal stability, different images of spots of red marker (Sharpie, Newell Brands, Atlanta, GA, USA) were performed with the initial parameters (capillary voltage: 0.8 kV, temperature of the ion transfer tube: 30 °C). This revealed that initial parameters should provide round, homogeneous, and plain spots between images performed on different days (Figure 2A), thus meaning that a stable signal could be obtained during and between analyses. Different capillary voltages were compared for the analysis of ULN: 0.6, 0.8, 1.0, and 1.2 kV, the former value being the upper limit recommended. Although the images revealed a higher concentration of the compound at the edge of the droplet, the intensities revealed that 0.8 kV was the most efficient capillary voltage, as illustrated in Figure 2B. Furthermore, different temperatures of the Heated Transfer Line were tested: 30, 100, 150, 200, 250, and 300 °C. As shown in Figure 2C, the different temperatures did not result in differences in the signal of ULN. Initial parameters of the DESI setup (capillary voltage: 0.8 kV, temperature of the ion transfer tube: 30 °C) were then kept for further development.

### 2.2. Fragmentation Characterization of ULN and ULN-d_6_

**Figure 3:**
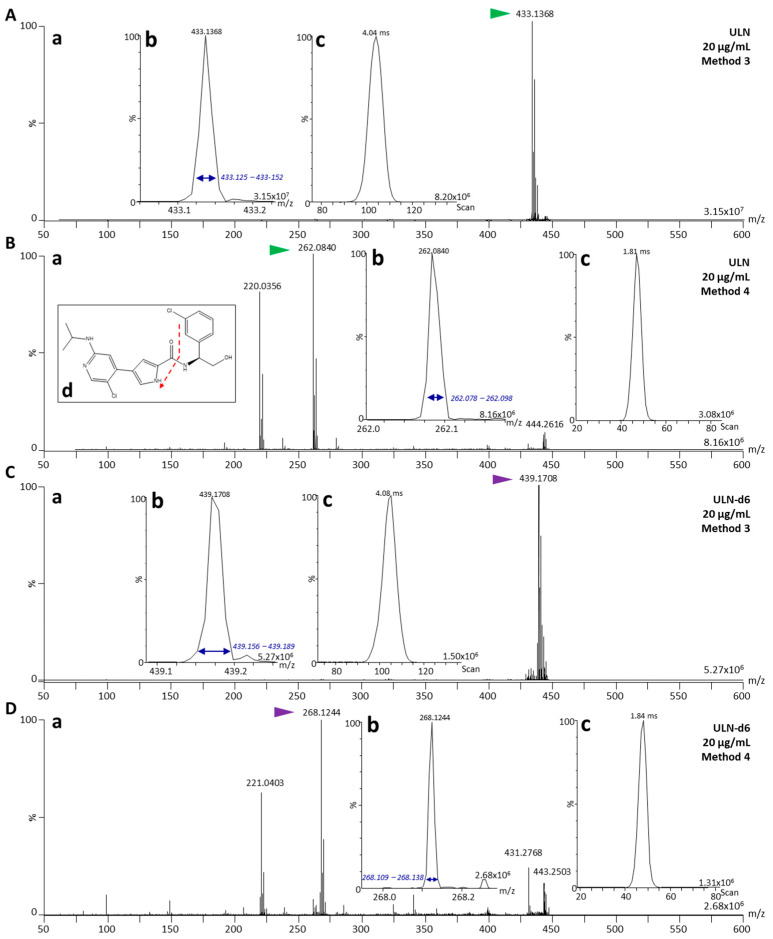
**Mass spectrometric (MS) characterization of the reference compound, ulixertinib (ULN), and its internal standard (IS), ULN-d_6_, as parent compounds or as fragmented compounds.** (**A**) Mass spectrum of the parent ion of ULN (*m*/*z* 433.14, (**A**(**a**))), with a zoomed view on the monoisotopic ion peak of ULN (green arrow, (**A**(**b**))) and the corresponding extracted ion mobilogram (XIM) with the mobility peak of intact ULN ((**A**(**c**)), extracted from the *m*/*z* range in blue arrows). (**B**) Tandem mass spectrum (MS/MS spectrum) of ULN (*m*/*z* 433.14, (**B**(**a**))), with a zoomed view on the monoisotopic ion peak of the fragment at *m*/*z* 262.08 (green arrow, (**B**(**b**))) and the corresponding XIM with the mobility peak of the ULN major fragment ((**B**(**c**)), extracted from the *m*/*z* range in blue arrows). The fragmentation pattern is given in (**B**(**d**)). (**C**) Mass spectrum of the parent ion of ULN-d_6_ (*m*/*z* 439.17, (**C**(**a**))), with a zoomed view on the monoisotopic ion peak of ULN-d_6_ (purple arrow, (**C**(**b**))) and the corresponding XIM with the mobility peak of intact ULN-d_6_ ((**C**(**c**)), extracted from the *m*/*z* range in blue arrows). (**D**) MS/MS spectrum of ULN-d_6_ (*m*/*z* 439.17, (**D**(**a**))), with a zoomed view on the monoisotopic ion peak of the fragment at *m*/*z* 268.12 (purple arrow, (**D**(**b**))) and the corresponding XIM with the mobility peak of the ULN-d_6_ major fragment ((**D**(**c**)), extracted from the *m*/*z* range in blue arrows). Analyses of parent compounds were acquired using Method 3 (selection of *m*/*z* 433 in the quadrupole and no fragmentation). Analyses of fragmented compounds were acquired using Method 4 (selection of *m*/*z* 433 in the quadrupole and fragmentation with a collision energy of 32 eV).

As previously demonstrated, IM-MS/MS analyses offer different advantages among DI methods for the development of drug quantification assays [41]. First IM-MS allows for (i) a gas-phase separation of drugs and endogenous compounds from the biological matrix for further post-acquisition signal filtering [2], and (ii) an increase of intensities and resolution of MS peaks thanks to ion focusing when using traveling-wave (TW)-IM-MS [2]. Second, MS/MS methods allow for a “dilution” of signals from endogenous compounds since fragments are detected at lower intensities and over a large mass range. This allows for a more specific distinction of the signal from the drug of interest [2,41]. Used with low-resolution quadrupoles (selection *m*/*z* range > 1 Da), both fragments of the targeted drug and corresponding IS can be measured at the same time, thus allowing for an efficient normalization, and partially correcting for signal instability that can be observed in DI-MS assays [41,48]. Indeed, it was previously shown that an overall stable signal would be necessary for the development of a robust quantification assay to be validated according to regulatory guidelines [41]. Therefore, it was evaluated if the new DESI sprayer would be robust enough for the development of IM-MS/MS assays for drug quantification following regulatory guidelines and the IM-MS/MS characterization of ULN was performed (Figure 1B). MS/MS methods require (i) high intensity reference standard fragments with isotopes that do not overlap or interfere with the signals from the monoisotopic ion of the IS fragments, and (ii) in case of a stable isotopically labeled IS, a high purity of labelling [48]. The fragmentation pattern of ULN and ULN-d_6_ were then first verified on Aquarray superhydrophilic glass slides using sub-stock solutions at 20 µg/mL. A profiling approach was adopted as it allows for the faster analysis of sample surfaces and no quantitative comparison was necessary for fragment characterization. The results are illustrated in Figure 3. ULN (*m*/*z* 433.14, Figure 3A) displayed a drift time (DT) of 4.04 ms. ULN-d_6_ (*m*/*z* 439.17, Figure 3C) displayed a DT of 4.08 ms. The fragmentation patterns indicated the presence of a major MS/MS fragment at *m*/*z* 262.08 with a DT of 1.81 ms (Figure 3B) and at *m*/*z* 268.12 with a DT of 1.84 ms (Figure 3D) for ULN and ULN-d_6_, respectively. As we formerly observed for other compounds and expected for the present ones, mobility peaks for fragments of ULN and ULN-d_6_ were sharper than for parent ions. Besides being the most abundant, the fragment pair *m*/*z* 262/268 was the only one displaying a ∆*m*/*z* of 6, thus preventing from any interference between the isotopes of ULN and the monoisotopic peak of ULN-d_6_.

Therefore, these fragments were further used for subsequent steps of the development of the quantification assay.

### 2.3. Signal Determination and Optimization on Tissue Sections

We further evaluated the signal of the compounds in the biological context for a rapid quantification method, i.e., on tissue sections from mouse brain and using a profiling approach. Further parameters directly influencing the sample-dependent desorption were tested to optimize the ion signal (Figure 1B).

**Figure 4:**
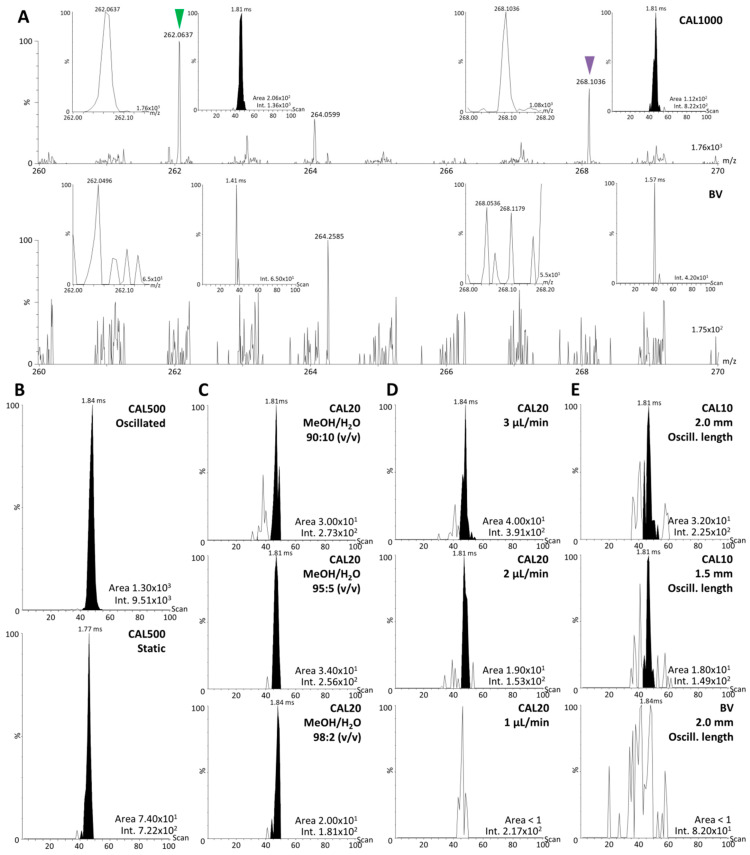
**Optimization of ulixertinib (ULN) signal during desorption electrospray ionization (DESI) analyses of mouse brain sections.** (**A**) Specificity of ULN DESI signal in tandem MS spectra between a CAL1000 sample (ULN spiked at 1000 ng/g on mouse brain section) and a blind value (BV) sample (blank mouse brain section). Insets show zoomed views on ULN and ULN-d_6_ peaks and related extracted ion mobilograms (XIM). (**B**) XIM from CAL500 samples showing signal improvement using spray oscillation versus static spray. (**C**) XIM from CAL20 showing signal optimization using different MeOH/H_2_O ratios (i.e., initial ratio: 98:2, 95:5, 90:10). (**D**) XIM from CAL20 showing signal optimization using different solvent flow rates for spraying. (**E**) XIM from CAL10 samples showing optimization of ULN signal by increasing oscillation length and enabling a better detection of ULN at the targeted lower limit of quantification (10 ng/g). Related area and intensities are given on the bottom right of each spectrum and XIM.

First, the highest calibration point (upper limit of quantification—ULOQ, CAL1000) of the intended quantification assay was deposited on tissue as previously described [2]. We first verified that the selected fragments of ULN and ULN-d_6_ did not interfere with fragments of endogenous compounds. The comparison between a blind value sample (tissue section with no standard) and a CAL1000 sample indicated that no interfering signal was present in the vicinity of ULN and ULN-d_6_ (Figure 4A). However, as expected and described previously [1], carry-over was detected after batch analyses of different CAL samples, showing the need for a regular cleaning process to avoid biased results from lower CAL samples.

Further optimization steps were then conducted in order to approach the targeted lower limits of quantification (LLOQ) of 10 ng/g (CAL10) or the next higher concentration level (20 ng/g, CAL20).

A first attempt of signal optimization consisted of performing the profiling analysis with an oscillation motion of 1.5 mm (see Section 4 and Section 4.6) in order to cover a larger area of tissue for the analysis. The results indicated a significant increase of the signal of the standards when tested in CAL500 (>15-fold), (Figure 4B). In order to solve the issue of possible carry-over, a cleaning of the DESI cone and the Heated Transfer Line was performed before all batches of analyses, with these two elements remaining mounted on the instrument, using a tissue paper soaked with MeOH/H_2_O 1:1 (*v*/*v*). Further tests were performed on CAL20 to improve the stability of the spray and detect CAL10 by changing parameters one by one, namely optimization of (i) the fine sprayer positioning, (ii) the gas flow, (iii) the solvent ratio, (iv) the solvent flow, and (v) the oscillation area. Changing the fine positioning of the sprayer and the gas flow did not show any improvement. Regarding the MeOH/H_2_O solvent ratio, an increased signal could be obtained for ULN in CAL20 using a 95:5 (*v*/*v*) ratio versus a 98:2 (*v*/*v*) ratio, while lower MeOH ratios led to lower ULN signals (Figure 4C). A slightly higher solvent flow rate of 3 µL/min showed a slight improvement compared to 2 and 1 µL/min (Figure 4D). Using the optimized parameters (initial sprayer position and gas flow, spray oscillation with a length of 1.5 mm, MeOH/H_2_O 95:5 (*v*/*v*) as solvent with a 3 µL/min solvent flow rate) allowed to detect ULN in CAL10 and increasing the oscillation length from 1.5 to 2.0 mm further improved the ULN signal (Figure 4E). It was therefore aimed to develop a quantification assay with an LLOQ of 10 ng/mL, using the final parameters (initial sprayer position and gas flow, spray oscillation with a length of 2.0 mm, and MeOH/H_2_O 95:5 (*v*/*v*) as solvent with a 3 µL/min solvent flow rate).

### 2.4. Analytical Batches for Calibration

With the parameters optimized as described above, it was aimed to verify whether the source could provide satisfactory parameters for the creation of calibration curves in terms of linearity, precision, and accuracy with the LLOQ defined at 10 ng/g. According to regulatory guidelines defined by the US Food and Drug Administration (FDA), the European Medicines Agency (EMA), and the International Committee for Harmonization (ICH) [1,49,50,51], calibration curves should be computed using the simplest possible regression model and the selected model should be reproducibly fitting between batches. Precision between replicates should be <20% CV (coefficient of variation) for the LLOQ and <15% CV for all other calibration points, and accuracy of the back-calculated concentration of calibration points should be within a range of ±20% bias for the LLOQ and ±15% bias for all other calibration points. Obtaining calibration curves that fall within these ranges can be considered the ultimate proof of the reliability of an ion source for the development of quantification assays. Two batches were performed on different days in order to define whether calibration curves would meet regulatory guideline criteria. As an additional criterion of quality, a determination coefficient (R^2^) above 0.98 was defined as a satisfactory value for calibration curve linearity.

**Figure 5:**
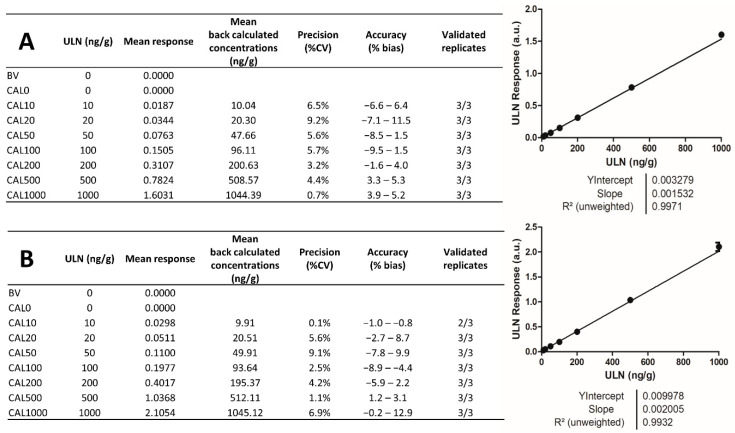
Analytical batches using triplicate analyses of calibration points. Accuracy and precision data with calibration curves obtained for (**A**) first batch and (**B**) second batch.

As mentioned above, the HP DESI sprayer and Heated Transfer Line tube should be cleaned before each batch of analyses to eliminate carry-over from previous analysis batches. It was observed that the desorption impacts shape and direction, and slight position changes of the Heated Transfer Line after cleaning could result in considerable variations in signal intensity. Together with the desorption pattern verification, three critical repositioning parameters were defined after source cleaning, before starting any batch: (1) the Heated Transfer Line angle, (2) the height of the stage after source cleaning (Z axis), and (3) the alignment of the desorption impact with the Heated Transfer Line. Before any batch of analyses, (i) a cleanup of the DESI cone and Heated Transfer Line was performed, (ii) the desorption pattern was checked, (iii) the above-mentioned positions were precisely readjusted based on the signal at *m*/*z* 443.24 obtained from water-sensitive paper (see Section 4 and Section 4.7), and (iv) LLOQ (CAL10 in our context) samples were analyzed to validate these readjustments. We observed that CAL10 could always be detected with a mobility peak area higher than 50. For further data processing, a threshold of 25 was set as minimum acceptable peak area for mobility peak detection during data extraction. We observed that the definition of this threshold also permitted to exclude peaks corresponding to noise at the basis of mobility peaks obtained with automatic peak selection. Using these parameters, the two analytical batches were performed with biological triplicates of each calibration point (Figure 5). According to regulatory guidelines defined by the FDA, EMA, and ICH [49,50,51], these batches displayed satisfactory linearity, as well as good precisions and accuracies of the different CAL levels. These results suggest that, as an ion source, the new HP DESI sprayer meets the required performance for the development of quantification assays according to regulatory guidelines.

## 3. Discussion

While presenting the advantages of DI-MS of low sample input and preservation of histological context, DESI-MS levels up the asset of rapidity of analysis in tissue sections. However, robust hardware setup was necessary to implement this instrumentation into the panel of MS approaches used for drug quantification following regulatory guidelines (FDA, EMA, ICH). In the present article, we evaluated the performances of new DESI enhancements, the HP DESI Sprayer and the Heated Transfer Line for the development of future rapid and sensitive quantification assays of the ERK inhibitor ULN in tissue sections. The HP DESI Sprayer was designed to address some of the drawbacks of the previous designs that affected the robustness of the spray and the Heated Transfer Line was designed to enhance the transfer of ions to the mass spectrometer. Initial tests indicated that the HP DESI Sprayer displayed strong stability over days. Although applying high temperatures to the Heated Transfer Line did not lead to signal enhancement in this study, future applications with other compounds may inform if this parameter is important for drug quantification. Additionally, optimization of spray and instrument parameters in the context of this assay allowed us to dramatically increase the ion signal of the compounds of interest and to perform pre-validation batches that reproducibly validated an LLOQ of 10 ng/g, whilst maintaining the accuracy and precision values that were acceptable within the criteria of regulatory guidelines. Although the present results suggest that the instrumentation is valid for the development of DESI-MS assays for drug quantification following regulatory guidelines, the long-term stability of the source will have to be evaluated to define its suitability for routine analyses. Among the hardware elements that need to be investigated, the nozzle and the emitter appear to be most critical, because the desorption properties strongly depend on these two parts. Slight deterioration of any of these two parts might have important consequences on assay performances, and a fine observation of the integrity of these elements might be necessary on a regular basis. However, since the metal emitter is incorporated in a plastic cartridge (see Section 4 and Section 4.6, Figure 7A(b)), its microscopic observation is difficult. Therefore, direct replacement may be recommended when a deterioration is suspected. Our experience indicated that slight changes in the desorption pattern and fine positioning can lead to >10-fold variation of signal intensity, thus possibly hampering the determination of the LLOQ and the validity of the developed assays. For targeted applications, it will be necessary to define an adapted procedure for system suitability tests. In the present study, we suggest (i) to verify the desorption pattern, (ii) to adjust fine positioning parameters and (iii) test the sensitivity of the source before starting any analytical batch. In this context, measuring the signal of LLOQ samples using a validated method for a drug of interest appears to be the most appropriate procedure to ensure that the system meets the sensitivity requirements for quantification.

**Figure 6:**
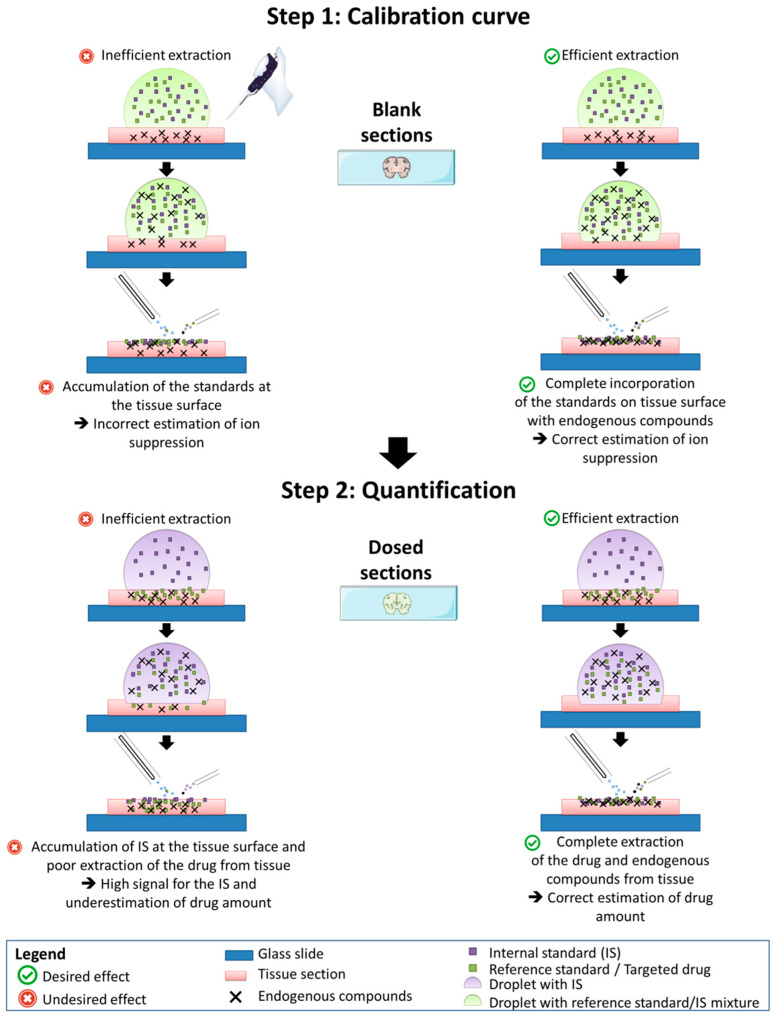
Possible artifacts during quantification of drugs from tissue sections induced by inefficient extraction.

It is important to note that in the present study, we only focused on analytical aspects in order to define if the new DESI setup provides the requested performances of an ion source for drug quantification in tissue sections. In terms of assay development, additional steps will be necessary to define that the chosen sample preparation method is adequate for drug quantification, without creating artefacts. Dedicated methods will be necessary to verify the proper extraction of drugs from sections of dosed tissues. It was formerly demonstrated that the presented sample preparation method was ideal for the extraction of mebendazole from tissue sections using MeOH/H_2_O 1:1 (*v*/*v*) as a solvent mixture, as confirmed by comparison of MALDI-IM-MS/MS with liquid chromatography (LC)-MS/MS results [2]. However, using alternative solvents such as tert-butyl methyl ether led to interacting artefacts and underestimation of drug concentrations [41]. For the generation of calibration curves, standards and IS are deposited on tissue sections, while for dosed tissue sections only the IS is deposited on the tissue sections and ULN is extracted from the tissue. Inefficient extraction in dosed tissue would result in an underestimation of the amount of ULN, as illustrated in Figure 6. Great care must be taken in the choice of solvent to ensure that extraction of drugs from tissue sections is optimal, and strategies must be developed to prevent artefacts from excessive accumulation of the IS on the surface of dosed tissues. Finally, method validation will necessitate to develop dedicated quality control (QC) samples to evaluate mimetic recovery (QC MIM), i.e., the yield of solvent-based extraction from dosed tissues [1]. Similar to profiling assays, the same precautions should be observed in DESI imaging for drug quantification, especially because approaches to solvent-based deposition of compounds could lead to significant artefacts in extraction of drugs from dosed tissues [1]. DESI imaging mode would provide the ultimate and powerful combination of spatial resolution and in situ quantification. Besides improvement for robustness, the High-Performance DESI Sprayer was designed to obtain a sharper spray plume for higher spatial resolution in DESI imaging. However, spatial resolution is often balanced with signal quality and intensity in imaging MS. Adequate sample preparation and analytical settings will have to be finely optimized to reach the needs in terms of LLOQ and histological resolution, depending on the selected pharmacological application.

## 4. Material and Methods

### 4.1. Chemicals

MS-grade H_2_O, organic solvents, and formic acid (FA) were purchased from Biosolve Chimie SARL (Dieuze, France). ULN and ULN-d6 were provided by BioMed Valley Discoveries (Kansas City, MO, USA). The purity of the two compounds were 99% and 98.26%, respectively. The structure of ULN is provided in Figure 1A.

### 4.2. Standard and Internal Standard Solution Preparation

ULN and its IS, ULN-d_6_, were weighed and stock solutions were prepared at 1.60 and 0.64 mg/mL in acetonitrile (ACN)/H_2_O 1:1 (*v*/*v*), respectively. A sub-stock solution of ULN was prepared at 20 µg/mL in MeOH/H_2_O 1:1 (*v*/*v*). From the sub-stock solution, calibration standard solutions (CAL) of ULN on seven non-zero levels were prepared with serial dilution of ULN stock solution from 2000 to 20 ng/mL in MeOH/H_2_O 1:1 (*v*/*v*), (Table 1). A sub-stock solution of the IS, ULN-d_6_, was also prepared from the IS stock solution in MeOH/H_2_O 1:1 (*v*/*v*) to reach 20 µg/mL. For each CAL level, the final solution to deposit on tissue (dilution mix) was prepared by mixing the corresponding ULN CAL solution and the IS sub-stock solution. The concentrations in these dilution mix solutions were 200 ng/mL of ULN-d_6_, and from 0.705 to 70.7 ng/mL for ULN (Table 1).

### 4.3. Deposition of Rhodamine C, Leucine Enkephaline, and Stock Solutions of Ulixertinib on Aquarray Superhydrophilic Glass Slides and Array Design

Aquarray DMA Slides G-dd-202 slides (Aquarray GmbH, Eggenstein-Leopoldshafen, Germany) containing 32 × 10 circle spots with 1414 µm diameter each were used for instrument calibration and an initial compound characterization. For instrument calibration, 3 µL of leucine enkephaline (Waters Corporation, Milford, MA, USA) at 400 ng/µL in ACN/H_2_O were deposited on a spot and analyzed by DESI-MS/MS with the parameters described below, defined after optimization. Acquisitions for mass calibration were performed in static profiling with oscillation, in TOF-MS/MS and sensitivity modes, over 60 s. To test the signal stability of the DESI-MS (Figure 1B), rhodamine C was deposited onto Aquarray spots by marking the surface using a red marker (Sharpie, Newell Brands, Atlanta, GA, USA). For ionization optimization and IM-MS/MS characterization of ULN and ULN-d_6_ (Figure 1B), 1 µL of the sub-stock solution described above was deposited onto Aquarray spots.

Because the current design of Aquarray slides does not include an array to localize the deposited spots, a grid was created in Adobe Illustrator CS2 (Adobe, San José, CA, USA) to guide the deposition of solutions (provided as Supplementary File S1) and displayed in Figure 8C. This grid was further used to guide the deposition of compounds on tissue sections.

### 4.4. Tissue Sectioning

For CAL samples, a female NGS untreated mouse aged 10 weeks and weighing 25 g was euthanized and the brain was dissected and snap-frozen in liquid nitrogen. Frozen brain was divided longitudinally in two hemispheres. From the whole two hemispheres, 10 µm-thick serial sections were made using a Leica CM 1950 UV cryostat and stored at −80 °C before sample preparation. All killings for organ removal were performed according to German Laws for Animal Protection and approved by the Institutional Review Board and the responsible animal welfare officer of the Deutsches Krebsforschungszentrum (DKFZ) (internal reference number DKFZ374).

### 4.5. Standard Deposition on Tissue Sections

Serial sections of one mouse brain were used for each analytical batch and deposition of standards was performed in the same histological region for each section (e.g., cortex). It is noteworthy that although sagittal tissue sections were used for these developments, coronal mouse brain sections (Figure 1B) could also be used, providing that deposits can be performed in similar regions between samples of the same analytical batches. This permits to obtain a minimal histological and molecular heterogeneity between samples to analyze. The deposition of solutions on tissue was described previously [2] (Figure 1B). From each dilution mix solution of ULN CALs, 1 µL was deposited onto a tissue section and three up-and-down pipetting motions were performed in order to extract endogenous compounds from the tissue in the deposition area. The solution deposition reproducibly created 3-mm diameter spots, that were systematically measured with a ruler as a quality control step. Since the deposit diameter, the thickness of the tissue sections, and the density of brain tissue are known, a concentration in ngstandard/gtissue can be calculated [2]. The final tissue concentrations of ULN at each CAL level are given in Table 1 and the final IS concentration on tissue was 28.3 ng/g.

### 4.6. Mass Spectrometric Analyses

The analyses were performed with a SYNAPT G2-Si instrument (Waters Corporation) consisting of an orthogonal acceleration (oa)-quadrupole (Q)-ion mobility (IM)-time-of-flight (TOF) mass spectrometer equipped with an enhanced DESI source and controlled using MassLynx^TM^ v4.1 (Waters Corporation).

**Figure 7:**
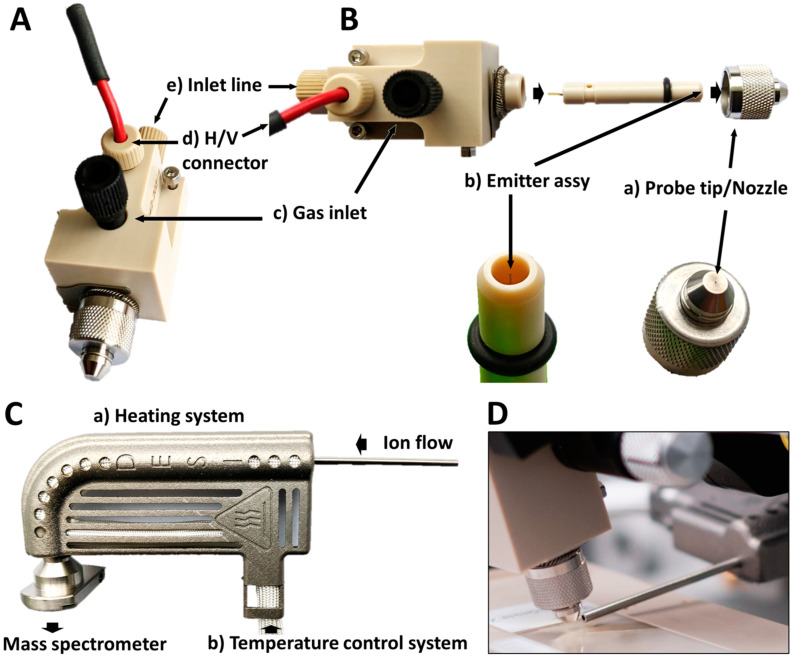
**Architecture of the High Performance (HP) DESI Sprayer and the Heated Transfer Line.** (**A**) Outer view and (**B**) inner view of the HP DESI Sprayer showing the location of the emitter and the critical connections (H/V, gas and fluid inlet line). (**C**) Heated Transfer Line showing the heating system and the position of the temperature control system. (**D**) Complete setup installed on the mass spectrometer.

The HP DESI Sprayer (available for DESI XS and here mounted on a Prosolia (Prosolia, Purdue, IN, USA) source) that was used for this study deviates from the previous model [46,47] in a few fundamental ways (Figure 7): (i) use of a metal electrospray ionization (ESI) emitter (Figure 7B(b)) rather than a glass emitter to assist in robustness of spray, (ii) embedding of the metal ESI emitter behind a narrow orifice in the probe tip/nozzle (also called cone below) (Figure 7B(a)), which protects the emitter from exogenous damages, (iii) flow of a nebulization gas through the orifice (Figure 7B(c)) helping to focus the spray and producing a sharper spray plume as compared to previous DESI sprayers, as well as an improved resolution, and (iv) ground connection of the sprayer orifice (Figure 7B(d)), producing less charging of tissue sections.

The different relative positions of the sprayer were as follows:

(i) 4 mm from the sample surface (z height: +1 mm), (ii) 3 mm from the MS inlet tube (y distance: 0 mm), (iii) 80° of inclination (10° to the sample normal), (iv) sprayer centered with the inlet tube (x offset: −0.5 mm).

Together with the HP DESI Sprayer, a prototype of Heated Transfer Line was also installed and tested (Figure 7C). It consisted of the previous model of ion collection tube [46,47] covered with a heating system (Figure 7C(a)) and monitored by an external control system (Figure 7C(b)). The ion transfer tube was placed 0.25 mm from the sample surface. The complete setup built on the mass spectrometer is presented in Figure 7D.

The solvent mixture flow was done with a nanoAcquity ultraperformance (UP)LC system (Waters Corporation) mixing MeOH and H_2_O with a MeOH proportion from 95% to 98%. The solvent flow was set from 1 to 3 µL/min. The motion of the sample stage was controlled by Omnispray software version 2.1.0.2 (Prosolia Incorporated, Purdue, West Lafayette, IN, USA). Two profiling approaches were used: static mode [47] and oscillating mode. The static mode consisted of positioning the DESI sprayer at the middle of the deposit without any further motion during the analysis. The oscillating mode consisted of moving the sample on the X axis through a defined length and during a defined time corresponding to the time of analysis.

The instrument was used in “resolution” mode (“W” mode) [11] and calibration was performed in TOF-MS/MS mode using leucine enkephalin deposited on Aquarray slides, as described above. For analytical development, the instrument was used in the IM mode. IM permits to obtain higher resolution and more intense peaks [2] thanks to ion focusing within the mobility cell, and to perform LC-MS-like data integration using mobility data. We previously demonstrated that integration of mobility data is essential for the validation of drug quantification assays [41] using DI-IM-MS/MS methods. The previously described IM-MS and MS/MS parameters [2] were used (i) for the analysis of the parent compounds (selection of specific parent ion using the Q followed by IM separation before MS detection, referred as Method 3) and (ii) for the analysis of fragments (selection of specific parent ion using the Q followed by collision-induced fragmentation at 32 eV and subsequent IM separation of the fragments before MS detection, referred to as Method 4). Detailed parameters are displayed in Table 2.

The quadrupole low mass (LM) resolution was set to 4.4 arbitrary unit (a.u.), to allow the selection of ULN and the ULN-d_6_ without losing sensitivity [2].The standard energy value for fragmentation (32 eV) was verified to be optimal for ULN, i.e., to produce intense MS/MS fragments and weak intensity peaks of remaining parent ions.

DESI-imaging was used with High Definition Imaging (HDI) v1.4 (Waters Corporation) to test for signal stability (Figure 1B). Method 3 and a motion speed of 600 µm/s were used. Spectra were acquired every 0.5 s, resulting in a pixel size of 300 µm.

### 4.7. Desorption Pattern and Mass Spectrometric Signal Evaluation

**Figure 8:**
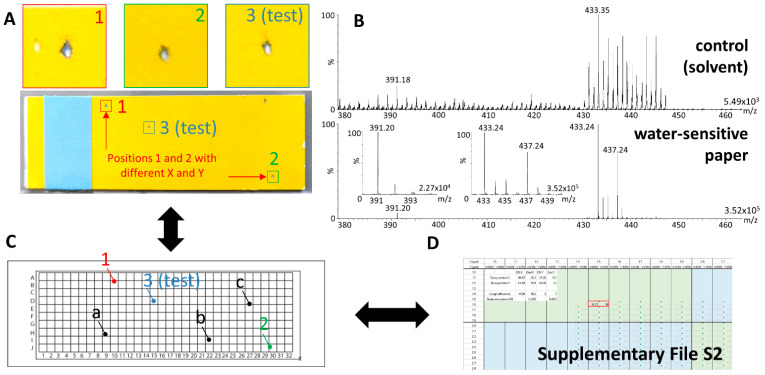
**Desorption pattern evaluation, mass spectrometric signal adjustment, and sample position localization.** (**A**) Typical desorption impact patterns using the High-Performance DESI Sprayer. Teaching points in 1 and 2, and a test point in 3 are created and respective x and y positions reported in Omnispray; (**B**) mass spectrometric signal obtained from water-sensitive paper with zooms on *m*/*z* 433.24 and *m*/*z* 391.19; (**C**) teaching and test x and y positions are reported in a grid and positions from the grid reported as well as positions of spots in samples (a, b, c); (**D**) Omnispray and grid x and y positions from teaching points are reported in an Excel file converting x and y grid positions into x and y Omnispray positions (Supplementary File S2). The precision of x and y Omnispray positions of the test point is verified in the Excel file as described in the standard operating procedure in the first table of Supplementary File S2; x and y Omnispray positions of samples are obtained by reading their equivalence from their x and y grid positions in the Excel file.

Since the desorption characteristics largely impact the analytical performance of DESI, it should be precisely verified before any analytical batch. For this purpose, water-sensitive paper (Syngenta, Basel, Switzerland) was used: it changes color from yellow to blue on contact with water or solvent and to white on mechanical compression, e.g., by a focused gas stream. We observed that a regular desorption beam which eventually led to successful analytical batches (see Results section, Section 2.4) created a small white impact surrounded by a thin blue layer in the direction of the mass spectrometer, as displayed in Figure 8A.

The desorption of the material constituting the water-sensitive paper created a strong signal for a parent ion at *m*/*z* 433.24 and at *m*/*z* 391.19 for a fragment ion, as shown in Figure 8B. Water-sensitive paper was then also used for signal optimization, especially for the adjustment of X and Z positions of the motion plate that are relevant for optimum signal intensity, as described below.

### 4.8. Localization of x-y Positions in Omnispray

The localization of the exact x-y position of sample spots is not intuitive because positions displayed by Omnispray are not reported physically on the x-y motion platform. On the other hand, variations in the position of the sprayer can change the position of the desorption beam, making it difficult to position the sprayer accurately in the direction of the x-y platform and thus the sample itself. In order to permit a fine positioning of the desorption beam, we created a grid that reports x-y positions displayed by Omnispray. The method relies on a teaching phase to correlate desorption areas with x-y positions displayed in Omnispray, using water-sensitive paper stuck on a histological glass slide (teaching slide). One teaching point was taken on the upper left corner (teaching point 1) and another on the lower right corner (teaching point 2) and the corresponding x-y position in Omnispray was noted (Figure 8A). The teaching slide was superimposed with a blank histological glass slide to mark the location of the teaching positions. This latter glass slide was overlaid with the grid provided in Supplementary File S1 and the coordinates for this grid were given (Figure 8C). Omnispray and grid coordinates from teaching points 1 and 2 were then documented in an Excel file as provided in Supplementary File S2 (Figure 8D) according to the standard operating procedure visible in slide 1. This converted every position of the grid into Omnispray x-y positions. For actual samples, positions could be reported on the grid and the excel file was consulted to know the Omnispray positions.

### 4.9. Data Processing

Mobilograms and MS spectra were extracted from MassLynx v4.1 and calibration curves were computed using Prism software version 5.01 (GraphPad, La Jolla, CA, USA). Recommendations were followed to report IM-MS measurements [52]: because IM is used here as a separation method and not for structural analyses, the DT are reported as IM data. Two-dimensional mobility maps (mass-per-charge (*m*/*z*) vs. DT maps) were obtained using Driftscope^TM^ version 2.9 (Waters Corporation). The previously described MobA method [2,41] was used for data extraction: the mobility peaks of the compounds of interest were first extracted from the regions of the mass spectra specific to each of the targeted compounds to obtain the specific extracted ion mobilograms (XIM). The obtained XIM were then automatically integrated to retrieve the peak areas using MassLynx software [2,11], and the normalized responses were calculated using the ratio of the ULN mobility peak area to corresponding IS mobility peak area. Data extraction was automated using the Chrotool feature from MassLynx to obtain the different XIMs (i.e., automatic extraction of the mobility data for the targeted specific mass range). DESI-MS images were analyzed using HDImaging v1.4 (Waters Corporation).

## 5. Conclusions

Overall, the present method development indicates that the HP DESI Sprayer is a robust enhancement to introduce DESI-MS among the panel of suitable instrumentation for the development of quantification methods validated according to regulatory guidelines. Additional steps are necessary for the development and the validation of in situ quantification assay according the regulatory guidelines, in order to define that the chosen sample preparation method will not generate artefacts in quantification. The full validation of DESI-MS methods would permit its use for quantification in the context of clinical trials using human samples. Finally, the major additional asset of DI-MS in pharmacology is the possibility to map exogenous compounds in tissue sections. Validation of quantitative DESI-MSI assays would allow to combine fine histological drug localization together with their precise quantification, for use in clinical trials.

## Figures and Tables

**Table 1:** Concentrations of the calibration standard (CAL) solutions of ulixertinib (ULN) in solutions and in spiked biological matrix.

Calibration Point	ULN CAL Solution Concentration (ng/mL)	ULN CAL Concentration in Dilution Mix (ng/mL)	ULN CAL Concentration in Tissue (ng/g)
CAL1000	2000	70.700	1000
CAL500	1000	35.300	500
CAL200	400	14.140	200
CAL100	200	7.070	100
CAL50	100	3.530	50
CAL20	40	1.410	20
CAL10	20	0.705	10
CAL0	0	0	0
Blind value (BV)	0	0	0

**Table 2:** Summary of the acquisition methods tested for the quantification of ulixertinib in tissue sections using DESI-IM-MS and DESI-IM-MS/MS.

Methods	Quadrupole	Collision Energy (eV)	Ion Mobility	Target ion ULN (*m*/*z*)	Internal Standard	Target ion IS/Control (*m*/*z*)
3	*m*/*z* 433	0	✓	433	ULN-d_6_	439/443 (rhodamine C from red marker)
4	*m*/*z* 433	32	✓	262	ULN-d_6_	268

## Data Availability

Data sharing contains in the main text and supplementary materials.

## Supplementary Materials

The following supporting information can be downloaded at: https://www.mdpi.com/article/10.3390/ph15060694/s1, Supplementary File S1: printable grid for position localization from Aquarray slides and tissue regions of interest; Supplementary File S2: Excel file converting Omnispray positions into grid (Supplementary File S1) positions.
