# Supplementary figures and images for: Analytical Performance Evaluation of New DESI Enhancements for Targeted Drug Quantification in Tissue Sections
Source: Pharmaceuticals (Basel). 2022 Jun 1;15(6):694. doi: 10.3390/ph15060694 (PMC9228120; doi:10.3390/ph15060694)

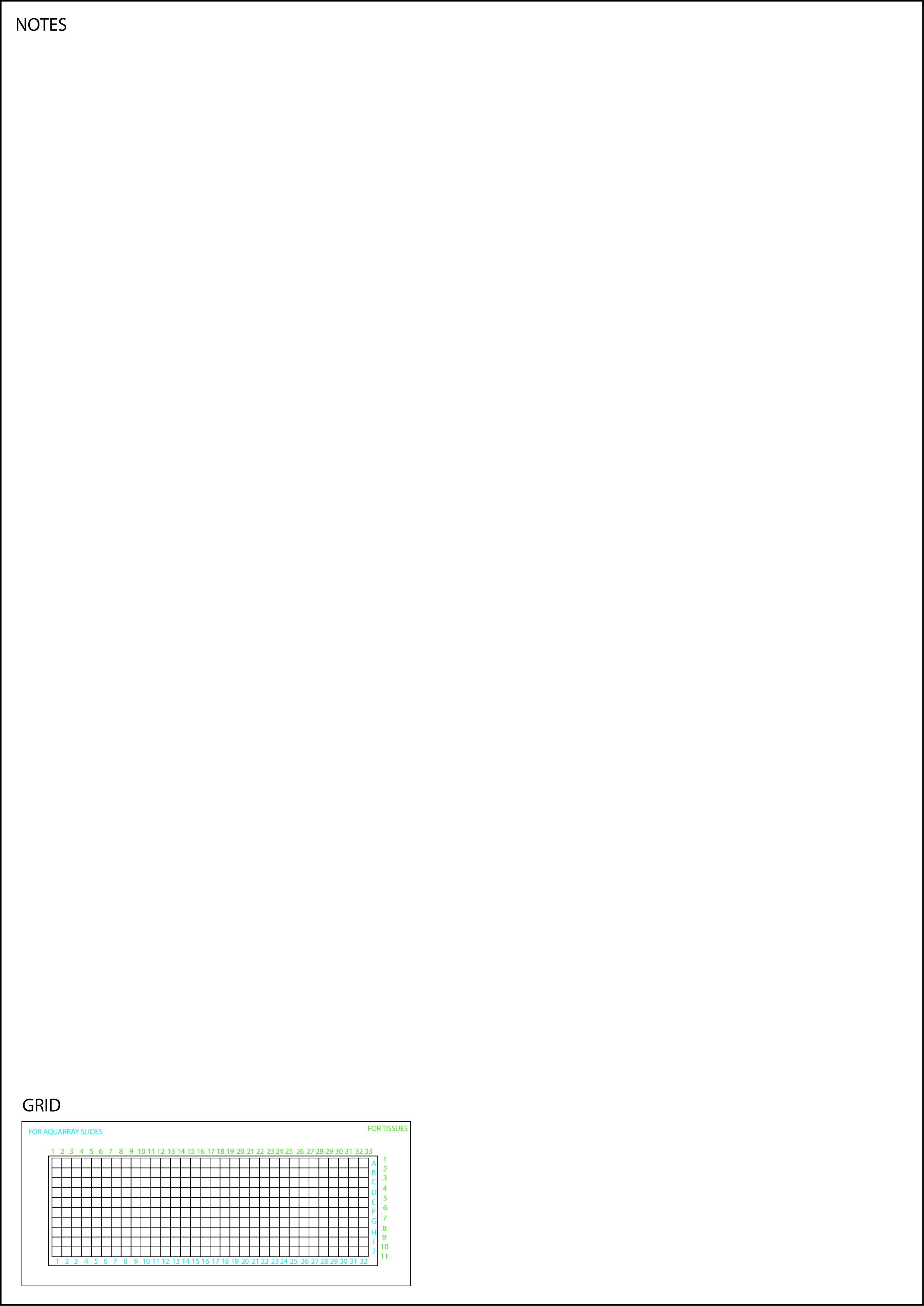

Supplement: Supplementary file 1 [file pharmaceuticals-15-00694-s001.zip › Supplementary_material_Proof/Supplementary File S1.tif]
